# The Personal and Contextual Contributors to School Belongingness among Primary School Students

**DOI:** 10.1371/journal.pone.0123353

**Published:** 2015-04-15

**Authors:** Sharmila Vaz, Marita Falkmer, Marina Ciccarelli, Anne Passmore, Richard Parsons, Tele Tan, Torbjorn Falkmer

**Affiliations:** 1 School of Occupational Therapy & Social Work, Curtin University, Perth, Western Australia, Australia; 2 School of Education and Communication, CHILD programme, Institution of Disability Research Jönköping University, Jönköping, Sweden; 3 School of Pharmacy, Curtin University, Perth, Western Australia, Australia; 4 Department of Mechanical Engineering, Curtin University, Perth, Western Australia, Australia; 5 Rehabilitation Medicine, Department of Medicine and Health Sciences (IMH), Faculty of Health Sciences, Linköping University & Pain and Rehabilitation Centre, UHL, County Council, Linköping, Sweden; University of Calgary, CANADA

## Abstract

School belongingness has gained currency among educators and school health professionals as an important determinant of adolescent health. The current cross-sectional study presents the 15 most significant personal and contextual factors that collectively explain 66.4% (two-thirds) of the variability in 12-year old students’ perceptions of belongingness in primary school. The study is part of a larger longitudinal study investigating the factors associated with student adjustment in the transition from primary to secondary school. The study found that girls and students with disabilities had higher school belongingness scores than boys, and their typically developing counterparts respectively; and explained 2.5% of the variability in school belongingness. The majority (47.1% out of 66.4%) of the variability in school belongingness was explained by student personal factors, such as social acceptance, physical appearance competence, coping skills, and social affiliation motivation; followed by parental expectations (3% out of 66.4%), and school-based factors (13.9% out of 66.4%) such as, classroom involvement, task-goal structure, autonomy provision, cultural pluralism, and absence of bullying. Each of the identified contributors of primary school belongingness can be shaped through interventions, system changes, or policy reforms.

## Introduction

Existing literature on school belongingness is fragmented across educational, psychological, health promotion and sociological fields [1]. Various terms such as, identification with school [2], relatedness [3,4], community [5], school membership [6], and connectedness [7] have been used interchangeably to refer to school belongingness. These terms encompass various domains including social experiences of an environment or relationship; feelings or attitude states; and associated behaviours [8–10]. School belongingness in the current study is defined as *“…*the extent to which students feel personally accepted, respected, included and supported by others in the school social environment” [11]. Such a conceptualisation of school belongingness extends beyond enrolment at school, to mean that students have established social bonds amongst themselves and with teachers, and perceive the rules governing schools as important and protective [6].

The question of whether an individual’s perception of school belongingness varies as a function of factors such as gender, disability, and household socio-economic status (SES) is compelling because of adverse outcomes (such as mental health problems, dropping out of school) that are associated with school belongingness. Evidence of the influence of student gender [12–15], disability [16,17] and household-SES [18,19] on school belongingness is mixed. Student personal attributes, such as, perceived competence [20–22], coping skills [23], motivational goals for schooling [24–27], and school-related activity participation [2,28–30] have been linked to school belongingness. Family factors such as, parental education and expectations from their child [31], family functioning [32], social support [33,34], and involvement in schooling [35,36] have also been positively associated with school belongingness.

Relationship and classroom management dimensions [27,37–43] have dominated the school belongingness research; with developmental dimensions of the classroom, such as, task-goal orientation [44,45], autonomy-support [42,43], cultural and diversity acceptance, and absence of bullying [46–48] identified as potential determinants. Few studies have considered the combined contribution of personal and contextual factors [49,50], despite the notion that school belongingness is a product of the interaction between the individual and the environment, and considered to be malleable and responsive to features of the school environment. Evidence on the hypothesized relationship between school type (private or public) and school belongingness is unsubstantiated mainly due to measurement issues, with existing population-based investigations inferring low perception of school belongingness from truancy levels, instead of directly measuring the construct [51,52].

Although several studies have focussed on factors associated with school belongingness in secondary school students [14,40,50,53–59]; fewer studies have focussed on the determinants of primary school belongingness [60,61]. Existing evidence, however, suggests that different factors may influence school belongingness at different year levels. Thus, to date, we have a limited understanding on whether factors associated with belongingness in primary school continue to predict belongingness once students transition to secondary school.

In recent years, school belongingness has also gained currency among educators and school health professionals as an important independent determinant of mental health, not only in typically developing adolescents, but also in students with disabilities [1,37,62–67]. School belongingness has also been linked to various educational outcomes, such as, school attendance [68], academic performance [69], and school completion [70]. School belongingness however has mostly been investigated as an antecedent or mediator of student outcomes, and not as an outcome in its own right [54]. This trend has been attributed to societal valuing of discrete outcomes, such as emotional and behavioural health, academic grades, dropping out of school, truancy, or drug use, over psychological attributes such as school belongingness. Given the detrimental effects of adolescents dropping out from school prematurely [65,71], schools and communities face the challenge of not only ensuring that students continue to *‘belong in school’*, but also of ‘trying to reconnect’ those who feel disconnected from school [37,72].

The majority of large scale investigations on school belongingness have excluded students with disabilities [65,66] and are limited to school students in the United States of America (US) [1]. More research is needed to substantiate the role of school belongingness in inclusive education practices [73–75], and to validate current research findings in other countries. By understanding factors associated with school belongingness, education leaders and teachers can facilitate the inclusiveness of school environments.

Drawing on the existing knowledge base, the current study aims to bridge the gap in the literature about the factors associated with primary school belongingness, and present the most influential personal and contextual factors, using a non US sample of primary school students with, and without disability.

## Methods

### Aims and objectives

The current study describes the most significant personal and contextual contributors of school belongingness, using a sample of 12—year old primary school students, enrolled in the final year of study at mainstream primary schools. The study is part of a larger longitudinal study investigating the factors associated with student adjustment in the transition from primary to secondary school [76,77]. In the current paper, cross-sectional data from 395 students, parents and teachers enrolled in the final year of primary school were used. All students were enrolled in a regular school in the educational districts of metropolitan Perth or other major city centres of WA. Students were categorised into the disability group if they were reported by a primary caregiver to have a medical diagnosis or a chronic ill health condition that impacted on their functioning, and attended over 80% of the school hours per week in a regular setting; with support provided as required. Ethics approval was obtained from Curtin University Health Research Ethics Committee in Western Australia (WA) (HR 194/2005). At all times, informed written consent was obtained from school principals, parents, teachers, and written assent was acquired from students.

### Data collection procedure

Survey questionnaires were administered to all participants, in the second semester (Terms 3 and 4) of the final year in primary school (classes 6 or 7). Administration guidelines were developed to minimise bias.

### Data collection instruments

Tables 1, 2 and 3 present an overview of the instruments used to measure the key factors identified in the literature as being associated with school belongingness, including covariates, student personal factors, and contextual factors (family and school context).

**Table 1:** Overview of covariates and student personal factors considered for inclusion in the school belongingness model.

	Factor	Instrument/ main source	Purpose	Rater	No of items or domains and meaning of total score	Psychometric properties (if needed—addition references to substantiate psychometrics if available)
**Covariates**	**Age Gender Presence/ absence of disability and type of disability**	Drawn from the Indicators of Social and Family Functioning Instrument Version-1 (ISAFF) [122] and Australian Bureau of Statistics surveys	Demographic profile of the sample to match the data to normative data	Parent/ Guardian	6-items	
**Student personal factors**	**Perceived Competence**	Self-Perception Profile for Adolescents [123]. Domains: academic competence; athletic competence; peer acceptance competence, physical appearance competence	Measures student perceived competence in various domains of functioning.	Student	5-domainsHigher score = higher competence	Cronbach’s α ranges from. 78 to.90 in populations of students with learning disability and behavioural disorders [123]. Considerate convergent, discriminant, and construct validity substantiated in equivalent US and Australian samples [124–126]. Discriminant validity among secondary school typically developing students, students with learning disability and behavioural disorders has been substantiated previously [127].
	**Coping skills**	Short form of the Adolescent Coping Scale (ACS) [128]. 3 coping styles: non-productive, problem solving, and reference to others.	Measures the usage and helpfulness of coping strategies in general and specific situations.	Student	3-coping styles: higher score = better coping style.	Cronbach’s α ranges from. 50 (reference to others) to. 66 (non-productive coping). Test-retest reliabilities range from. 44 to. 84 (Mean *r* = .69) [128]. Validated in Australian samples [128].
	**Motivational orientation for schooling**	Inventory of School Motivation (ISM) [129,130]. Domains: Task goals: (Mastery) task and effort motivation, Ego goals (Performance): competition and social-power motivation, Social solidarity goals: affiliation and social concern motivation, Extrinsic goals praise and token reward.	Assesses information on the goals students adopt for schooling	Student	8-domains. Higher score = higher related motivation	Cronbach’s α ranges from. 53 to.81. Adequate content, construct validity and test-reliability substantiated in cross-cultural studies [25,26,130–132]
	**Expectations for schooling**	Personal expectations. Perception of teachers & parent/guardian expectations of schooling [133].	Assesses students expectations for schooling and their perception of their parents’ and teacher’s expectation.	Student	3-items	Cronbach’s α is. 91. [133].
	**Mental health functioning**	Strength and Difficulties Questionnaire (SDQ) [27,40]. Domains: emotional, conduct problems, hyperactivity/inattention, and peer relationship scores	Brief screener of children and adolescents’ behaviours, emotions and relationships.	Parent/Guardian	1-domain of overall mental health functioning Higher score = worse mental health functioning (pro-social skills not included in total score)	Cronbach’s α ranges from. 70-.80 [134]. Adequate discriminate and predictive validity [27,40]. Widely used in clinical populations [135] and with adolescents with intellectual disability [136,137].

**Table 2:** Overview of contextual family factors considered in the school belongingness model.

Contextual factor: Family factors	Factor	Instrument/ main source	Purpose	Rater	No of items or domains and meaning of total score	Psychometric properties (if needed—addition references to substantiate psychometrics if available)
**Family demographics**	**Background**: Structure, Family income, time spent in paid employment, parents’ educational background	Obtains information about the family’s demographic factors	Parent/Guardian	6-items	Adapted from [86,122] [138] (ANZSCO) [139].
**Perceived social support from one’s family**	Multidimensional scale of perceived social support (MSPSS) [140,141]	Measures subjective perceptions of social support adequacy from the family	Student	1-domainHigher score = higher support	Cronbach’s α for the total scale is. 91. Subscale α = .90 to. 95. Test-retest reliability coefficient of. 85. Adequate factorial & concurrent validity have been documented [140,141].
**Family functioning**	Overall general functioning subscale of the McMaster family assessment device (FAD) [32,142]	Measures the perception of “how the family unit works together on essential tasks”	Parent/Guardian	1-domainHigher score = worse functioning	Cronbach’s α for the total scale. 86. 1- week, test-retest reliability = .71 Split-half coefficient = .83Good construct validity [32,142]
**Parental expectations of schooling for child**	Expectation of schooling [133]	Rates parental expectations for their child’s future success. Options ranged from primary level qualifications through to post-graduate degrees	Parent/Guardian	1- item	Developed by researcher [133]
**Parental involvement in education**	Multidimensional assessment of family involvement [143]. Domains: Home-School Communication, Home-Based Involvement, School-Based Involvement	Assesses parental involvement in their child’s education	Parent/Guardian	3-domainsHigher score = greater parent involvement	Cronbach’s α range from. 84 to.91. Validity reported to be adequate [143].

**Table 3:** Overview of contextual school and classroom factors and outcomes considered in the school belongingness model.

Contextual factor: School and classroom factors	Factor	Instrument/ main source	Purpose	Rater	No of items or domains and meaning of total score	Psychometric properties (if needed—addition references to substantiate psychometrics if available)
**School climate and adequacy of resources**	Type of school, services offered by school to address child’s needs. Information on the school sector, post code, number of students enrolled in each school, and organisational structure at each school was obtained from Department of Education and Training, WA records.	Obtain demographic details of the school	Parent	5- items	Developed by researcher [144,145]. Cronbach’s α is. 92.
**Student’s perception of the classroom environment**	The Middle School Classroom Environment Indicator (MSCEI) [146] Subscales: Student cohesiveness, Ease, Autonomy, Task-Orientation, and Involvement subscales Single items on bullying and cultural/disability tolerance [47,48,147,148]	Measures students’ perception of the psychosocial features of the classroom environment. The scale is drawn from works of contemporary classroom environment research and the growing body of knowledge on middle schooling [72,149,150]	Student	7-domainsHigher score = better classroom environment	Cronbach’s α ranges = .63 to.81. Overall factor structure, discriminate validity, and alpha reliability of MSCEI are robust [47,48,147,148].
**Parents’ perceptions of general invitations for involvement offered by their child’s school**	Parent Involvement Scale [118]	Measures parents’ perceptions of general invitations for involvement offered by their child’s school	Parent/Guardian	1-domain Higher score = higher involvement	Cronbach’s α = .78 and construct validity of this measure has been confirmed factor analysis [118].
**School belongingness**	**School belongingness**	Psychological Sense of School Membership (PSSM) Goodenew [11,57], Overall total score on 18-items (with a five-point response format)	To measure the degree to which a student feels accepted and included within the school	Student	1-domainHigher score = greater belongingness	Cronbach’s α = .80.Test-retest reliability = 0.78 (4-week interval) [151] and. 56 and. 60 for boys and girls (12-month interval) [64]. The total PSSM scores correlate positively with school success [11,57], lower levels of depression [64], and lower levels of anxiety [72]. PSSM has been shown to discriminate between groups of students predicted to be different in terms of their sense of belonging in school [11].

### Sample size estimation

Sample size was estimated based on the assumption that there would be approximately 15 independent variables in the final regression model. In order to have power of. 90 (*β* = 0.1) and *α*-value of. 05 (Type I error), a sample size of 215 was adequate to detect a small to moderate effect size of 0.1 (Sample Size Program: PASS) [78]. A sample size of 69 children with disabilities in the comparison group was adequate to detect a between group effect difference of. 47 or larger; when *α* was at. 05 and power set at. 8 (β = .2).

## Data Analysis

Analyses were conducted using the Statistical Package for the Social Sciences (SPSS Version 20) and Statistical Analysis System (SAS Version 9.2) software packages. Using the estimation maximization (EM) algorithm and Little’s chi-square statistic, the data were found to be missing completely at random, with the probability level set at 0.05 [79,80]. Standard guidelines recommended by instrument developers were followed to replace missing values in these questionnaires. In cases where guidelines were not available, missing values were replaced using mean value substitution [81]. Only small amounts of data were missing (< 2.5% at scale level), and independent samples *t*—tests confirmed that the profiles of those whose data were missing for various questions were similar to those who responded.

Descriptive statistics were run to summarise the profiles of study participants. In order to test for the effect of clustering of students (i.e., nesting of students in classes within schools) on their school belongingness scores, a Hierarchical Linear Model was fitted using the mixed procedure in SAS.

Pearson correlation coefficients were used to examine associations between independent variables (IV; student personal factors and contextual factors) and the dependent variable (DV; school belongingness score) prior to undertaking regression. Multiple linear regression models were thereafter fitted to describe the relations between the set of IVs (continuous and categorical) and the primary school belongingness score. Traditional regression summarizes the relationship between the DV and IVs by describing the mean of the response for each fixed value of the IV, using a function referred to as the conditional mean of the response [82,83]. Because linear regression exclusively focuses on the conditional mean, it can detract attention away from the properties of the whole distribution and thus fail to identify informative trends in the response distribution. The straightforward assumptions of a linear relationship between IVs and DV, and that the linear relationship increases smoothly across the range of the IVs, were tested by dividing each IV into quartiles [84]. Gender, disability status and household-SES levels were taken as fixed factors and each IV was regressed to the school belongingness score, using General Linear Model Analysis of Variance (ANOVA). In instances where the school belongingness score appeared to vary in a linear fashion across the three or four quartile categories of the IV; the IV was reverted to its original continuous scale. The final presentation of IVs thus varied as a function of whether their marginal mean estimates supported a consistent linear trend across the school belongingness score. Dummy variables were created to represent categorical IVs (personal, family and school contextual factors) incorporated into the regression models.

The assumptions of linear regression were tested by undertaking preliminary screening of the data through examination of residuals; examination of the scatterplot of residuals against predicted values; and testing for multivariate outliers [80]. No obvious pattern to the errors was detected through examination of the residual scatterplots. No multivariate outliers were found in any of the steps [80].

### Development of the model of student belongingness in primary school

A three-step process was followed to develop the model of primary school belongingness.

#### Step 1: Covariates

Linear regression models with interaction terms were fitted to test the influence of gender, disability, and household-SES on students’ school belonging scores. Interaction terms were dropped from the model if they were found not to be significant.

#### Step 2: Covariates + Identification of student personal factors and contextual factors added in each block

The covariates were added in Step 1 and stepwise backwards elimination was undertaken to identify the significant factors (*p* < .05) within student personal, family, and school contexts that were associated with belongingness in primary school.

#### Step 3: Rating the explanatory power of independent variables

The explanatory power of factors in blocks was assessed on the basis of how much each factor block added to the prediction of school belongingness, over and above that accounted for by the preceding block [85].

The order of entry of blocks into the analysis was:

Block 1: Covariates (gender, disability, and SES**)**;
Block 2: Student personal factors;
Block 3: Family factors; and
Block 4: School and classroom factors.

Output checks from Standard Multiple regression in SPSS that houses the Tolerance, Variance Inflation Factor (VIF) and the Collinearity Diagnostics output suggested multi-collinearity was not a problem [79]. Following convention, a *p*-value < .05 was taken to indicate a statistically significant association in all tests.

## Results

### Characteristic of the study’s sample

Cross-sectional data from 395 students, their parents and primary-school class teachers from 75 different primary schools distributed across metropolitan Perth and other major urban centres of Western Australia were used. Students were on average 11.89 years (SD = 0.45 years, median = 12 years). Boys constituted 47.3% (n = 187) of the sample and 22% (n = 87) were reported by a parent or primary care-giver to have a disability. The predominant disability/chronic health conditions included asthma (18.8%), auditory disability (15.9%), Attention Deficit Hyperactivity Disorder / Attention Deficit Disorders (14.5%), learning disability (11.6%), Autism Spectrum Disorders (10.1%), and cerebral palsy (8.7%). The majority of the students (58%, n = 224) were from mid-range SES households; followed by high-SES households (32.1%, n = 124) and low-SES families (9.8%, n = 38) [86].

### Testing the effect of clustering of students in classes on their school belongingness scores

The class-level Intra Class Correlation Coefficient (ICC) for the primary school belongingness was 4% which suggested that the contribution of clustering to the overall variance was small. Based on these findings we can confidently state that for the study’s sample, after adjustment for gender, disability, and household-SES, clustering appeared to have minimal effect on the relationships between the student personal factors and school belongingness scores. Hence, further analyses were undertaken at the level of the individual student.

### Predictors of primary school belongingness at the level of the individual student

Table 4 displays the unstandardized regression coefficients (B) and standard errors (SE), and the standardized regression coefficients (Beta), and R, R^2^, and adjusted R^2^ after entry of all variables. R was significantly different from zero at the end of each step. No significant interactions were found; so, interaction terms were deleted from the final models.

**Table 4:** Factors associated with belongingness in primary school (N = 395).

Model	Factor	Unstandardized Coefficients		Standardized Coefficients	*t*	Sig.	95% Confidence Interval for B	
		Β	SE	Beta			Lower Bound	Upper Bound
**Step 1 Covariates**	(Constant)	3.80	.06		59.08	<.001	3.68	3.94
	Girls	.09	.07	.06	1.21	.228	-.06	.23
	Disability	-.07	.09	-.04	-.78	.438	-.24	.10
	Low-Q SES household	-.08	.13	-.03	-.63	.528	-.33	.17
	High-Q SES household	.18	.08	.12	2.25	.025	.02	.34
	R = .157, R^2^ = .025 adjusted R^2^ = .014							
	*F* [4, 365] = 2.32, *p* = .057							
**Step 2 Covariates + student personal factors**	(Constant)	3.07	.23		13.19	<.001	2.62	3.53
	Girls	.13	.05	.09	2.44	.015	.06	.24
	Disability	.11	.066	.07	1.73	.085	-.02	.24
	Low-Q SES household	-.02	.09	-.01	-.24	.807	-.20	.16
	High-Q SES household	.01	.06	.00	.10	.921	-.11	.12
	Social acceptance competence^a^	.21	.05	.20	4.36	<.001	.11	.30
	Physical appearance competence^b^	.15	.04	.15	3.54	<.001	.07	.23
	Low-Q cope solve the problem^c^	-.52	.07	-.33	-7.86	<.001	-.65	-.39
	Non-productive coping^d^	-.02	.00	-.29	-7.28	<.001	-.02	-.01
	Affiliation motivation^e^	.14	.03	.20	5.12	<.001	.08	.19
	R^2^ Change = .471, R = .704, R^2^ = .495, adjusted R^2^ = .483, F change for R^2^ = 67.17, *p* < .001							
	F [9, 360] = 35.96, *p* < .001							
**Step 3 Covariates + student personal factors + family factors**	(Constant)	3.03	.23		13.14	<.001	2.58	3.48
	Girls	.11	.05	.08	2.09	.037	.01	.21
	Disability	.15	.07	.09	2.34	.020	.02	.28
	Low-Q SES household	.02	.09	.01	.18	.854	-.16	.19
	High-Q SES household	-.07	.06	-.05	-1.18	.239	-.19	.05
	Social acceptance competence^a^	.18	.05	.18	3.83	<.001	.09	.27
	Physical appearance competence^b^	.15	.04	.15	3.59	<.001	.07	.23
	Low-Q cope solve the problem^c^	-.49	.06	-.32	-7.68	<.001	-.62	-.37
	Non-productive coping^d^	-.02	.00	-.27	-7.16	<.001	-.02	-.01
	Affiliation motivation^e^	.15	.03	.21	5.58	<.001	.10	.20
	Trade Vs University expectations for child^f^	.22	.06	.16	3.88	<.001	.11	.33
	Low-Q school-based involvement by parent^g^	-.15	.06	-.10	-2.50	.013	-.27	-.03
	R^2^ Change = .030, R = .725, R^2^ = .525, adjusted R^2^ = .510, F change for R^2^ = 11.12, *p* < .001							
	F [11, 358] = 39.282, p < .001							
**Step 4 Covariates + student personal factors + family factors+ school and classroom factors**	(Constant)	1.74	.26		6.64	<.001	1.22	2.25
	Girls	.11	.05	.08	2.36	.019	.019	.19
	Disability	.14	.06	.08	2.47	.014	.03	.25
	Low-Q SES household	.01	.08	.00	.10	.920	-.14	.16
	High-Q SES household	-.05	.05	-.03	-.99	.323	-.15	.05
	Social acceptance competence^a^	.13	.04	.13	3.30	<.001	.05	.21
	Physical appearance competence^b^	.10	.04	.10	2.84	.005	.03	.17
	Low-Q cope solve the problem^c^	-.24	.06	-.16	-4.09	<.001	-.36	-.13
	Non-productive coping^d^	-.01	.00	-.22	-6.41	<.001	-.02	-.01
	Affiliation motivation^e^	.11	.02	.15	4.57	<.001	.06	.15
	Trade Vs University expectations for child^f^	.14	.05	.10	2.75	.006	.04	.23
	Low-Q school-based involvement by parent^g^	-.14	.05	-.09	-2.66	.008	-.24	-.04
	Classroom involvement^h^	.17	.04	.17	4.08	<.001	.09	.25
	Low-Q task goal orientation^i^	-.16	.06	-.11	-2.75	.006	-.27	-.05
	Autonomy provision^j^	.10	.04	.11	2.78	.006	.03	.17
	Low-Q parental invitation for involvement^k^	-.11	.05	-.07	-2.14	.033	-.20	-.01
	Cultural pluralism^l^	.17	.04	.17	4.73	<.001	.10	.24
	Disagree Vs Agree to being bullied^m^	-.15	.05	-.10	-2.99	.003	-.24	-.05
		R^2^ Change = .139, R = .815, R^2^ = .664, adjusted R^2^ = .648, F change for R^2^ = 24.29, *p* < .001							
		F [17, 352] = 40.93, p < .001							

NOTE: Social acceptance competence^a^ and Physical appearance competence^b^ measured using the Self-Perception Profile for Adolescents [123]; Low-Q cope solve the problem^c^ and Non-productive coping^d^—measured using the Short form of the Adolescent Coping Scale (ACS) [128]; Affiliation motivation^e^—measured using the Inventory of School Motivation (ISM) [129,130]; Trade Vs University expectations for child^f^—measured using the Expectation of schooling scale [133]; Low-Q school-based involvement by parent^g^ and Low-Q parental invitation for involvement^k^ measured using the Multidimensional assessment of family involvement [143]; Low-Q task goal orientation^i^, Autonomy provision^j^, Cultural pluralism^l^, Disagree Vs Agree to being bullied^m^—measured using the The Middle School Classroom Environment Indicator (MSCEI) [146]. Where variables are prefixed by ‘Low-Q’ or ‘High-Q’ this refers to the low or high quartile of the distribution (as described in the Methods).

#### Block 1

Demographic factors including gender, disability and household-SES accounted for 2.5% of the variability in primary school belongingness (F _(4, 365)_ = 2.32, *p* = .057). Girls (Beta = .08, *p* = .019) and students with disability (Beta = .08, *p* = .014) reported higher belongingness than boys, and their typically developing counterparts; respectively. No variability in primary school belongingness due to household-SES was documented.

#### Block 2

The addition of student personal factors enabled the model to explain 49.5% of the variability in school belongingness. The increment in the model’s predictive power was significant (R² change = .471, F statistic for R² change = 67.17, *p* < .001).

Five student personal attributes significantly contributed to the primary school belongingness model. Higher belongingness was associated with concurrent high levels of social acceptance competence (Beta = .13, *p* = .001), low physical appearance competence (Beta = .10, *p* = .005), and high social affiliation motivational goal orientation (*Beta* = .15, *p <*.001). The use of low-levels of problem-solving coping skills relative to the average problem-solving group (Beta = -.16, *p* < .001), and frequent use of non-productive coping strategies (such as, worrying, ignoring the problem at hand, and self-blame) (Beta = -.22, *p* < .001) was associated with lower school belongingness. Students’ perceptions of scholastic competence, the pursuit of academic goals for schooling, and their mental health functioning did not influence their school belongingness scores.

#### Block 3

With the addition of family factors in Block 3, the model’s predictive power increased to 52.5% (R² change = .030, F statistic for R² change = 11.12, *p* < .001). Students whose parents reported low school-based involvement (Beta = -.09, *p* = .008) were less likely to feel they belonged in school, while those whose parents expected them to secure a university degree were more likely to feel belongingness (Beta = .10, *p* = .006). Other parent factors, such as social support and parental self-efficacy did not significantly contribute to the model of school belongingness.

#### Block 4

School and classroom factors when added in Block 4, enabled the model to explain 66.4% of the variance in school belongingness (R² change = .139, F statistic for R² change = 24.29, *p* < .001). Students who perceived their classroom to have low level task-goal structure (Beta = -.11, *p* = .006) were less likely to belong when compared to their counterparts who reported average level task-goal structure. Positive associations were found between school belongingness and involvement in classroom activities (Beta = .17, *p <* .001); and belongingness to highly autonomous (Beta = .11, *p* = .006) and culturally pluralistic classroom environments (Beta = .17, *p* < .001). Students were less likely to feel belongingness if their parents reported that their teachers extended low invitations for parental involvement in their schooling (Beta = -.07, *p* = .033). Students who reported being bullied in primary school also reported lower concurrent school belongingness (Beta = -.10, *p* = .003).

## Discussion

The current study presents the 15 most significant student personal and contextual factors that explain two-thirds (66.4%) of the variability in 12-year old students’ perceptions of belongingness in primary school. Demographic variables, including gender and disability accounted for 2.5% of the variability in student belongingness. Student personal attributes explained the majority of the variability in school belongingness (47.1 out of 66.4%) followed by school factors (13.9%), and family factors (3 out of 66.4%).

Students who perceived themselves to be socially accepted by their peers, and adopted social affiliation goals for schooling had higher belongingness scores [25,26]. We draw on competence theories to explain these findings. Competence theorists would argue that an individual’s perception of competence is a result of success in an achievement context [22,87]. People gravitate to domains in which they perceive self-competence and avoid domains/activities with which they do not have a sense of accomplishment. Research suggests that those with low social acceptance competence are more likely to avoid peers, and consequently experience further rejection and less attachment to school [88–90]. Students who perceive themselves to be poorly socially accepted by their peers may also have poorer social and communication skills, and hence find it difficult to belong to a peer group [91–93]. Not being associated with a peer group could damage the individual’s perception of interpersonal competence further [94], which in turn could perpetuate lower perception of school belongingness.

To conclude that school belongingness is mainly dependent on personal attributes and abilities would, however, disregard the fact that the personal factors explaining school belongingness, i.e.; social acceptance, social affiliation motivation, perception of physical appearance, problem solving skills and coping strategies, can all be influenced by how well the school environment satisfies the student’s need for belongingness [95]. Previous studies show that perceived acceptance from peers enhances the student’s motivation to pursue pro-social goals; and that this is more likely to occur in school settings that encourage supportive relationships among students [95]. Furthermore, teachers’ interactions with students tend to influence the students’ views of each other. Hence, the results from the current study support the suggestion that classroom strategies that allow for numerous and positive interactions between teachers and students, and among students, can be useful mechanisms for fostering school belongingness [14]. Consistent with the findings of others [96–98], we found positive associations between physical appearance competence and school belongingness. This may indicate that an accepting school environment that addresses negative ideals of body image and creates a culture wherein an individual’s strength and character are valued, can foster a sense of belonging amongst its students [99].

Our findings substantiate past works on the unfavourable associations of non-productive coping strategies on student adjustment [100–102]. Primary school students who use non-productive coping strategies (e.g., worrying, ignoring the problem at hand, self-blame), and low levels of problem-solving coping strategies (e.g., working at a problem while remaining optimistic) were shown to be less likely to sense belonging in school. These findings highlight the need for primary schools to not only provide students with opportunities to problem-solve, but also to support those who display non-productive coping strategies. While infrequent use of problem-solving coping was detrimental to school belongingness, frequent use of problem-solving coping was not any more beneficial than average use. This could mean that there could be a threshold level, beyond which the add-on benefits of problem-solving coping on school belongingness are insignificant. Further research into this area is needed.

In the current study we found that girls reported higher school belonging scores than boys. This finding concurs with earlier investigations that report associations between gender, and ability of the student and teachers’ predilections [95]. The consistent reporting of higher school belonging in girls may be a signpost for teachers to reflect on how they interact with the boys in the classroom [95].

Several aspects of the classroom environment including task-goal structure; opportunities for student autonomy; classroom involvement; cultural pluralism; safety, and anti-bullying were each found to be significantly associated with school belongingness. The central role played by classroom task-goal structure and student autonomy warrants elaboration, especially in light of current evidence of the effects of reduced task-goal orientation and autonomy on student outcomes [103,104]. A non-linear relationship was found between student perception of the task-goal orientation of the classroom and their school belongingness scores. Students in highly structured classrooms did not report any greater belongingness than their peers in average level task-goal structured classrooms. Students’ perceptions that their classroom had low task-goal structure were detrimental to their sense of school belongingness. Classroom task-goal structure enhances determination and regulation amongst students by providing them with autonomy, time-management strategies, and choice over their work [1,43,84,105]. Our findings suggest that primary school teachers should use at least moderate level task-goal structured teaching strategies, have clearly defined classroom structure, well defined expectations, and transparent assignment structure in order to foster belongingness amongst their students.

The level of a student’s involvement in classroom activities was also associated with their sense of belongingness. It is likely that involving students in planning and shaping of learning activities in class, promotes autonomy and self-determination [4,43,106], and makes them more likely to abide by the norms and rules of the classroom [14,43]. This means that in order to nurture school belongingness, primary school teachers need to encourage students to discuss ideas in class, ask questions, and collectively solve problems as a group. It could be hypothesised that class room practices are dependent of the perceived school climate. It has been shown that a democratic school climate has greater impact on students’ belongingness that structural characteristics such as the size of the school, facilities and if the school is a private or public school [107]. A democratic school climate may allow for teachers to implement more of collaborative teaching strategies which in turn would result in more interactions between students, further enhancing school belongingness [95]. However, a democratic school climate should be evident also outside the classroom and create a school structure in which students partake in rulemaking, are allowed to express concerns about the fairness of existing rules and are encouraged to participate in free and open discussions and in organising school events.

Students who reported to be bullied in primary school also had lower belongingness scores when compared to their peers who were not bullied. This finding is a cause for concern; especially in light of the wealth of longitudinal research on the detrimental effects of bullying on the individual’s mental health and wellbeing [108]. The current study’s findings support the implementation of whole-of-school bullying interventions from an early age, much before students reach the final year of primary school [109].

An unexpected finding was the higher school belongingness scores in the subgroup of students with a disability. This finding is in contrast to a previous study [16], and suggests that students with mild disabilities report similar school belongingness levels as their typically developing peers, despite having lower grades and behavioural vulnerability. The higher belongingness scores among students with a disability in our study could be a result of selection bias, i.e**.,** inclusion into the study was restricted to students who attended regular classes for 80% of the school hours [110]. Furthermore, students with mild cognitive impairments reportedly have a bias towards choosing the most positive response option when presented with a Likert-type rating scale [111]. The risk of a positive bias is increased if the scale relates to subjective issues, as in the case of the measurement scales used in our study [112]. These results should therefore be interpreted with appropriate caution.

No variability in school belongingness due to household-SES was found. This finding differs from past US investigations that have reported positive associations between school disengagement (and lack of belongingness) and economic disadvantage among middle and older adolescents [15,19,113,114]. The absence of any moderating effect of household-SES on school belongingness could mean that in primary school, one’s social standing is neither beneficial, nor detrimental to perceived school belongingness. This could also be a measurement effect (i.e**.,** small size of lower household-SES group which could have made detection of between-group differences difficult) or a function of the study context (Australia Vs. US). Nonetheless, the current study’s results warrant longitudinal investigations that track students along the educational continuum, to determine whether there is a cut-off point at which the effects of social disadvantage on school belongingness emerge.

The current study’s findings also endorse the role of parents in early adolescence [115]. Similar to the findings of others [27,104], students in the current study were less likely to sense school belonging if their parents reported low-level involvement in their school-based activities (e.g., infrequent volunteering in classrooms and infrequent escorting children on trips), and had low scholastic expectations (i.e., did not expect them to go to university). Theorists would argue that parental endorsement of scholastic expectations and involvement in their child’s schooling are a form of social capital conducive for membership to the school setting [35,116–118]. However, parents’ involvement is also dependent on them feeling invited to participate in school matters; hence, the school has a responsibility to make parents feel a welcome part of the school community.

It is noteworthy that in the multivariate model; structural attributes of the school, such as mean school SES, sector, and organisational model of schooling, each failed to influence the students’ school belongingness scores. The absence of any significant association between school structural attributes and 12-year old Australian students’ perceptions of school belongingness could be a function of context, student age, or over-inclusion of students from higher SES backgrounds. Nonetheless, it is reassuring that factors amenable to change across the classroom setting have a more potent influence on 12-year old Australian students’ sense of school belonging than intractable physical attributes that are often difficult to change [56].

### Limitations

There were several methodological issues that impact of the rigor of this study, all of which have been discussed in a previous publication [110]. The study sample was drawn from metropolitan Perth and other major urban centres across Western Australia. Students from other regional, and remote populations, or other major metropolitan cities in Australia were not part of the sample; which limits the generalisability of the study’s findings. Despite extensive recruitment efforts, 70% of the schools declined to participate in the study, which may have introduced a possible bias. The composition of the study's cohort included 29% from Catholic Education schools, 47% from public (government) schools, and 24% from independent (non-government) schools. This composition is different to the profiles of students in primary schools across these education systems in Western Australia (15% Catholic Education, 72% public, and 13% independent schools respectively).

Also, only personal development dimensions of the classroom environment, such as goal structure, disciplinary and autonomy provision were considered in our statistical model [43–45,119,120]. To avoid circularity, we did not consider the influence of teacher support and classroom relationship dimensions such as cohesiveness and affiliation on school belongingness, as we considered these to also be components of school belongingness [14,27,54,121]. Inclusion into the disability sub-group was restricted to caregiver report; with students reported to be enrolled in a mainstream class for 80% of their week considered for inclusion. This means that the ability to generalise the findings of the current study to students with more severe disabilities may be limited. Also, the confounding effect of disability severity and comorbidity status on school belongingness was not accessed. Replication of the study findings in students from more diverse school settings (i.e., educational support units, separate schools) is needed.

Given the quantitative nature of the study’s design, we did not explore how students with a disability conceptualised school belongingness, and whether their perception differed from that of their typically developing peers. The cross-sectional nature of the data presented in this study means that no causality can be determined. From a methodological standpoint, there may be models with other predictors as plausible as the ones we have presented.

## Opportunities

It is encouraging that each of the influences we identified can be shaped by educators through implementation of whole-of-school and classroom-based interventions, and policy reforms in the various education systems.
